# PKCθ Regulates T Cell Motility via Ezrin-Radixin-Moesin Localization to the Uropod

**DOI:** 10.1371/journal.pone.0078940

**Published:** 2013-11-08

**Authors:** Judy L. Cannon, Francois Asperti-Boursin, Kenneth A. Letendre, Ivy K. Brown, Katy E. Korzekwa, Kelly M. Blaine, Sreenivasa R. Oruganti, Anne I. Sperling, Melanie E. Moses

**Affiliations:** 1 Department of Molecular Genetics and Microbiology, University of New Mexico School of Medicine, Albuquerque, New Mexico, United States of America; 2 Department of Pathology, University of New Mexico School of Medicine, Albuquerque, New Mexico, United States of America; 3 Department of Computer Science, University of New Mexico, Albuquerque, New Mexico, United States of America; 4 Department of Medicine, Section of Pulmonary and Critical Care Medicine, University of Chicago, Chicago, Illinois, United States of America; University of Iowa, United States of America

## Abstract

Cell motility is a fundamental process crucial for function in many cell types, including T cells. T cell motility is critical for T cell-mediated immune responses, including initiation, activation, and effector function. While many extracellular receptors and cytoskeletal regulators have been shown to control T cell migration, relatively few signaling mediators have been identified that can modulate T cell motility. In this study, we find a previously unknown role for PKCθ in regulating T cell migration to lymph nodes. PKCθ localizes to the migrating T cell uropod and regulates localization of the MTOC, CD43 and ERM proteins to the uropod. Furthermore, PKCθ-deficient T cells are less responsive to chemokine induced migration and are defective in migration to lymph nodes. Our results reveal a novel role for PKCθ in regulating T cell migration and demonstrate that PKCθ signals downstream of CCR7 to regulate protein localization and uropod formation.

## Introduction

T cells comprise the major effectors of immune responses: T cells assist B cells in antibody production and are critical to mediate cellular immunity for pathogen elimination. Prior to activation, naïve T cells circulate in and out of lymph nodes constantly surveying for antigen [1]. This surveillance is critical for T cell function, facilitating T cell interaction with dendritic cells carrying antigen from tissues. In the absence of activation, T cells continually circulate in and out of lymph nodes, maximizing the potential of T cell encounter with antigen. Regulation of T cell trafficking is also an important aspect of immune-mediated disease states, including immune rejection of tumors [2], cardiovascular disease [3], and autoimmune diseases such as diabetes [4].

T cell migration to lymph nodes is mediated by the chemokine receptor CCR7 ligation by CCL21 which leads to upregulation of the integrin LFA-1 and the induction of a characteristic migrating T cell morphology [5]. Migrating T cells form a leading edge and a trailing uropod which play distinct roles in cell motility: the leading edge senses migration cues and drives motility forward while the uropod is responsible for cell retraction [6]. It has been shown that T cells are much less sensitive to activation by TCR signals when the signals are delivered to the uropod rather than the leading edge, suggesting that these two regions serve separate functions [7].

Specific functions arising from the leading edge and uropod in a migrating T cell likely result from distinct protein localization within these regions. Chemokine receptors are enriched at the leading edge, while the microtubule organizing center (MTOC), along with actin regulatory proteins ezrin-radixin-moesin (ERM), concentrate at the uropod [8]. ERM proteins are responsible for localizing its interacting partners to the uropod, including CD43, CD44, and ICAM [9], [10]. The uropod is enriched in cytoskeletal and adhesive components that can contribute to the generation of forces that regulate T cell migration across endothelial layers and in tissue. The localization of proteins in migrating T cells is likely to be a key determinant in how a T cell moves.

While many cell surface ligands have been shown to be important in regulating T cell migration into lymph nodes and to inflammatory sites, relatively little is known about the intracellular signaling mechanisms that regulate migration. Recent studies have implicated signaling molecules downstream of T cell receptor signaling [11] as well as regulators of the actin cytoskeleton such as Rac GTPases and myosin IIA [12], [13], [14]. PKC proteins are important signaling mediators in many cell types including T cells, leading to changes in cellular proliferation, cytoskeleton organization, and differentiation [15]. PKCθ belongs to the novel PKC subfamily, activated by diacylglycerol (DAG) but not calcium [15] through phosphorylation at Thr538, Ser676, and Ser695 [16], [17]. In T cells, PKCθ is a key signaling mediator downstream of T cell receptor engagement leading to T cell survival and differentiation through activation of NF-κB, NFAT, and AP-1 [18], [19], [20]. Although several PKC family members are expressed in T cells, only PKCθ showed specific localization to the immunological synapse, and it is the only PKC known to be essential for IL-2 expression [21].

While protein localization is clearly important in regulating T cell function, PKCθ localization appears to play a particularly crucial role in T cells. PKCθ was the original marker for the immunological synapse (IS), which forms at the interaction site between a naïve T cell and an antigen presenting cell [22]. PKCθ localization at the IS is likely to be important for both downregulation of the TCR signaling cascade as well as creating symmetry in the IS [23]. Recent evidence shows that PKCθ moves away from the IS between a regulatory T cell and a target cell, demonstrating that differential PKCθ localization may be important in controlling effector versus regulatory T cell function [24].

Protein localization is critical for T cell motility. Members of the PKC family including PKCβ and PKCθ have been suggested to play a role in regulating T cell motility [25], [26]. However, studies implicating PKCθ in T cell migration used broad and non-specific chemical inhibitors to assay for the role of PKCθ. Thus, the specific role of PKCθ in controlling T cell motility remains unknown.

We sought to understand the role of the PKCθ signaling pathway downstream of CCR7 signaling in T cells. In our study, we identify for the first time a specific role for PKCθ in regulating T cell motility. We show that PKCθ is specifically localized in migrating T cells and is activated upon CCR7 ligation independently of the T cell receptor. PKCθ is required for normal localization of the MTOC and ERM proteins to the migrating T cell uropod. We also show a role for PKCθ in regulating in vivo migration of T cells to lymph nodes. These results demonstrate that PKCθ plays a role downstream of CCR7 in driving T cell migration, and reinforces the importance of PKCθ localization in T cell function, including during T cell migration.

## Results

### PKCθ Specifically Localizes to the Uropod in Migrating T Cells

**Figure 1:**
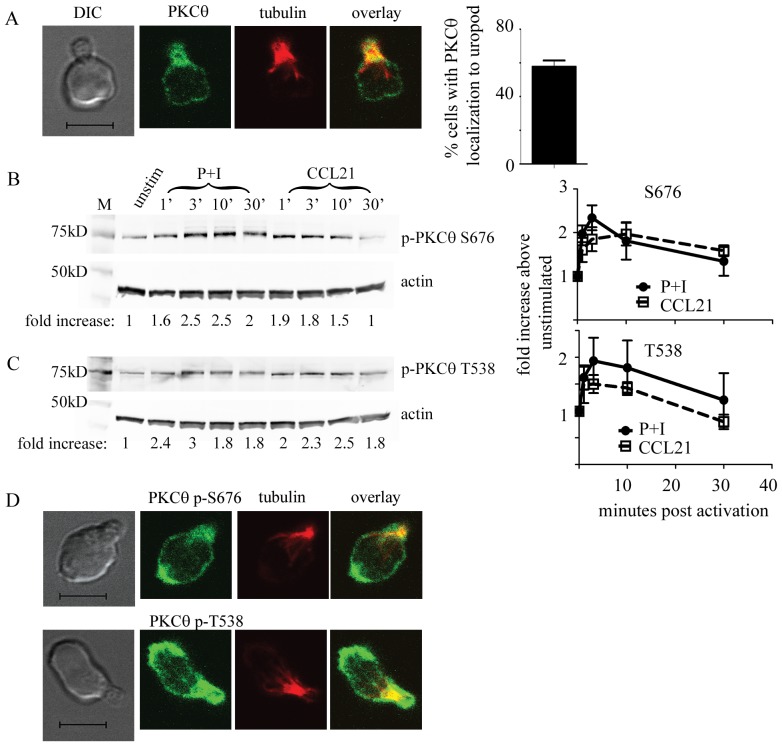
PKCθ is activated by CCR7 signaling and shows specific localization within migrating T cells. (A) WT T cells from C57Bl/6 or C57Bl/6 Ly5.1 mice were activated with 300 ng/ml CCL21 for 10 minutes, adhered onto Poly-L-lysine coated coverslips, fixed, and processed for immunofluorescence. Cells were stained with anti-α tubulin antibody (in red) to mark the uropod and anti-PKCθ (green). The scale bar indicates 5 µm. Quantitation of percentage of migrating cells showing PKCθ localization to the uropod is shown on the right. 3 experiments with at least 50 cells in each experiment were quantitated and the average shown. (B and C) Purified T cells were activated with either 50 ng/ml PMA and 500 ng/ml ionomycin (P+I) or 300 ng/ml CCL21 as marked for the indicated time points. Cells were lysed and analyzed on SDS–PAGE, transferred onto PVDF membranes and blotted with anti-actin and anti-phospho-PKCθ S676 (B) or anti-phospho-PKCθ T538 (C) antibodies. Signals were quantified using the Licor Odyssey and data shown is representative of at least 3 independent experiments. Fold increase in phosphorylated PKCθ was normalized to the level in the unstimulated WT condition. (D) WT T cells were activated with 300 ng/ml CCL21, adhered onto Poly-L-lysine coated coverslips, fixed, and processed for immunofluorescence. Cells were stained with anti-tubulin antibody (in red) to mark the uropod, and also phospho-PKCθ S676 (top), or phospho-PKCθ T538 (bottom) (both in green). The scale bar indicates 5 µm.

PKCθ is a critical player of the PKC family in T cells through its specific localization and ability to activate the NF-κB pathway [18]. While much work has shown the localization of PKCθ upon T cell activation, little is known about the localization and function of PKCθ in migrating T cells independent of TCR activation. As PKCθ localization is crucial to T cell function in other contexts, we asked whether PKCθ might also localize specifically in migrating T cells. We investigated PKCθ localization in migrating T cells by treating naïve wild type T cells with the CCR7 ligand CCL21 to induce a motile T cell morphology. We then fixed and processed the migrating T cells for immunofluorescence and detected the localization of PKCθ using anti-PKCθ antibodies. We also used anti-alpha tubulin antibody to detect the location of the microtubule organizing center (MTOC) which marks the uropod in migrating T cells [27]. While alpha tubulin is not localized exclusively to the MTOC, we have previously shown that it is enriched at the MTOC, showing staining at a spot that indicates the location of the MTOC [28]. We find that PKCθ shows a specific localization to the uropod within migrating T cells (Fig. 1A). PKCθ (green) localizes near the MTOC (red), with yellow areas showing co-localization between tubulin and PKCθ. 57% (+/−9%) of all cells with uropods showed PKCθ localization in the proximal third of the cell labeled by anti-tubulin staining (Fig. 1A, quantitation on right). This data agrees with published data showing that other PKC isoforms PKCδ and PKCβI also localize near the MTOC in multiple cell types, including T cells [29].

### PKCθ Phosphorylation is Increased by Chemokine Signaling in T Cells Independently of TCR

Upon T cell receptor stimulation, PKCθ activation is reflected by phosphorylation at specific serine and threonine residues. Serine 676 (S676) and threonine 538 (T538) are key sites within PKCθ that are phosphorylated in response to TCR activation [16]. If PKCθ acts downstream of CCR7, we hypothesized that CCR7 stimulation should induce PKCθ phosphorylation. We assessed whether chemokine receptor signaling could induce PKCθ phosphorylation at both T538 and S676 by treating primary murine T cells with CCL21 to activate CCR7, then determining the level of PKCθ phosphorylation at S676 and T538 by western blotting. Both antibodies were shown to be specific for PKCθ as indicated by a lack of reactivity towards PKCθ-deficient T cells (data not shown). As a control, we treated T cells with PMA and ionomycin, which activates the PKCθ pathway by mimicking TCR signaling. We found, as expected, that PMA and ionomycin treatment increased PKCθ phosphorylation at both S676 (Fig. 1B, quantitation on right) and T538 (Fig. 1C, quantitation on right) as early as one minute and could last up to 30 minutes. Similarly, we found that CCL21 treatment of naïve T cells also led to an increase in phosphorylation of both S676 (Fig. 1B) and T538 (Fig. 1C). Both phosphorylation at S676 and T538 increased at 1 minute, was maximal at 3 minutes post CCL21 treatment, and returned to near baseline by 30 minutes with phosphorylation at S676 staying more elevated. Phosphorylation at S676 was significantly increased (p<0.05) when treated with PMA+ionomycin at 1 and 3 minutes, and with CCL21 at 30 minutes when compared to baseline. While a similar trend was seen for T538 phosphorylation, the level of T538 phosphorylation was not significantly increased with either treatment. The level of PKCθ phosphorylation in response to CCL21 was comparable to that seen with PMA+ionomycin treatment, increasing 1.5 to 2.5 fold above unstimulated levels.

As phosphorylated PKCθ is likely to be the pool responsive to chemotactic cues, we asked whether phosphorylated PKCθ also localized specifically within migrating T cells. To do this, we treated purified primary murine T cells from wild type mice with CCL21 and then processed the cells for immunofluorescence. We detected the localization of the phosphorylated forms of PKCθ using the phosphorylation specific antibodies to S676 and T538 (Fig. 1D). Interestingly, while a pool of phospho-PKCθ S676 remained at the T cell uropod like that seen for total PKCθ (Fig. 1A), phosphorylated S676 PKCθ also showed localization to the leading edge of the migrating T cell (Fig. 1D). This leading edge localization was also seen for phosphorylated T538 (Fig. 1D). These data show that PKCθ localization and activation is responsive to chemokine independent of TCR signaling.

### PKCθ Regulates T Cell Migration

**Figure 2:**
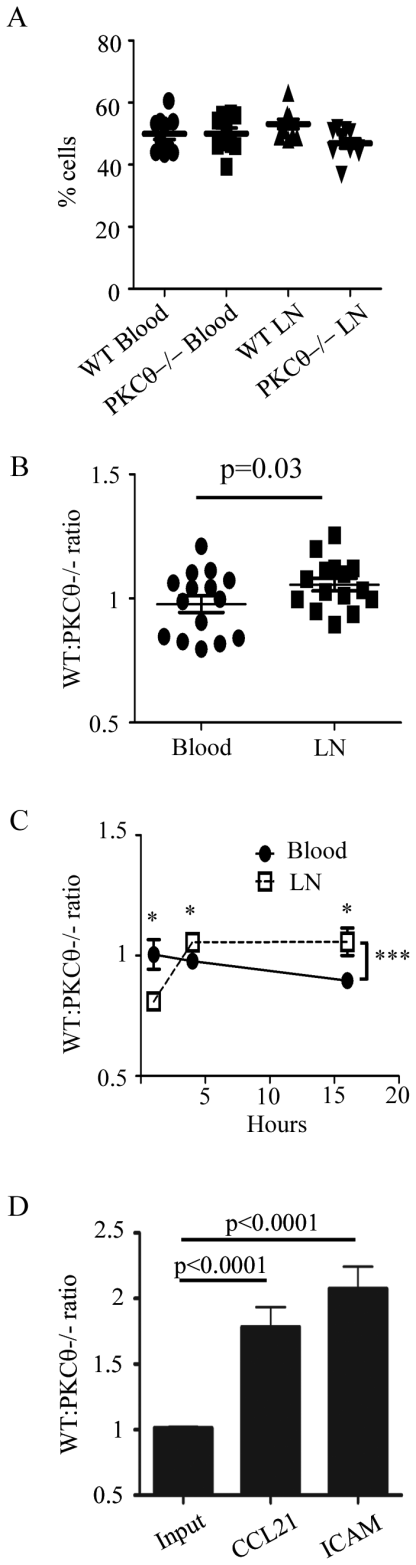
PKCθ is required for T cell migration to lymph nodes. T cells were isolated from C57Bl/6 or B6.PKCθ−/− mice and each population stained with different concentrations of CFSE (D), or 0.5 µM CFSE and 0.5 µM PKH26 (A,B,C), combined, and adoptively transferred into recipient C57Bl/6 Ly5.1 mice (A,B,C) or added to the top of a Costar 3 µm Transwell insert (D). (A,B) Cells were allowed to migrate for 4 hours, and the ratio of migrated CD4+ cells was analyzed using flow cytometry. Percentage of each population in each organ (A) or as a ratio (B) is shown. (B) Data shown are the average of 3 independent experiments with 6 mice each, each dot represents an individual mouse. Significance was determined by a paired student’s t-test. (C) Adoptively transferred cells were allowed to migrate for 1, 4, and 16 hours, then blood and lymph nodes were harvested and percentage of cells in each organ analyzed by flow cytometry and the ratio of CD4+ WT: PKCθ−/− T cells calculated. Data are the average of 3 independent experiments and error is the SEM. Significance was determined using the unpaired student’s t-test with * indicating p<0.05. The *** p = 0.0007 indicates a 2-way ANOVA analysis of the difference between the ratio of WT and PKCθ−/− T cells in the blood vs LN. (D) The bottom of the inserts contained 300 ng/ml CCL21 or coated with 6 µg/ml ICAM-1. Cells were allowed to migrate for 4 hours, and the ratio of migrated cells was analyzed using flow cytometry. Data shown are the average of 3 independent experiments and error is the SEM. Significance was determined using the unpaired student’s t-test with p value shown.

Our results showing specific activation of PKCθ and localization to the uropod upon activation by CCR7 stimulation suggests that PKCθ may regulate T cell migration downstream of CCR7. CCR7 is required for T cell entry into lymph nodes via the high endothelial venules (HEV) as well as T cell motility within lymph nodes [5]. We determined whether T cell migration to lymph nodes was affected by the absence of PKCθ using a competitive in vivo homing assay. We differentially labeled purified primary T cells from wild type or PKCθ−/− mice with fluorescent vital dyes CFSE or PKH26, then combined the two populations and adoptively transferred the combined WT and PKCθ−/− T cells into recipient mice expressing the congenic Ly5.1 marker. We allowed for migration into lymph nodes, then harvested the blood and lymph nodes of recipient mice and assessed the ratio of WT versus PKCθ−/− T cells in each organ by CFSE or PKH26 staining with anti-Ly5.2 (donor T cells) and anti-CD4 antibodies to identify transferred T cells using flow cytometry. We first identified the percentage of each cell population in both blood and lymph nodes (Fig. 2A). We also calculated the ratio of WT to PKCθ−/− T cells after we normalized the percentage of each population to the injected population (Fig. 2B, see materials and methods for more details).

We assessed migration at 1, 4, and 16 hours post transfer. We found that in the blood, the percentage of WT and PKCθ−/− T cells recovered was approximately 50%–50% for all time points observed, resulting in a ratio of close to 1 for WT: PKCθ−/− T cells at each time point assayed (Fig. 2B,C). Interestingly, we found that at 1 hour post injection, more PKCθ−/− T cells were found in lymph nodes than WT T cells. In contrast, at 4 and 16 hours, we saw fewer PKCθ−/− T cells in lymph nodes compared to WT T cells (Fig. 2C). At 1 hour, the ratio of WT: PKCθ−/− T cells was 1 for the blood and 0.8 for lymph node, indicating a 20% increase in PKCθ−/− T cells in the lymph node relative to WT T cells. However, at 4 hours and 16 hours, we saw approximately 10–20% increase in the number WT cells compared to PKCθ−/− T cells (Fig. 2C). We found a similar migration effect whether we assayed total T cells, CD4 or CD8 populations, or naïve CD62Lhi populations (data not shown).

Because in vivo migration to lymph nodes combines effects on multiple aspects of T cell motility, we wanted to determine whether PKCθ had direct effects on T cell migration downstream of CCR7. To isolate CCR7 induced migration, we used an in vitro transwell assay. The transwell filter contains an upper chamber which separates cells by pores of 3 µm size from the lower chamber which holds chemokines and adhesion ligands. We differentially labeled wild type and PKCθ-deficient T cells with different concentrations of the fluorescent dye CFSE, then combined the differentially labeled populations and added them in approximately equal ratio (1∶1) to the upper chamber. We allowed the cells to migrate to CCL21, then analyzed the ratio of migrated cells in the bottom of the transwell compared with the ratio of input cells. In agreement with our hypothesis, PKCθ-deficient T cells showed approximately 2-fold defect in migration to CCL21 compared to wild type T cells (Fig. 2D). We also determined whether PKCθ might have effects on migration to LFA-1. We found that PKCθ−/− T cells migrated less to ICAM-1 compared with WT T cells (Fig. 2D). We found no additional defect when we combined CCL21 with ICAM-1 (data not shown). Our in vivo and in vitro migration data show that PKCθ can affect T cell migration directly in response to CCL21 via CCR7 signaling.

### PKCθ Effect on T Cell Motility within Lymph Nodes

**Figure 3:**
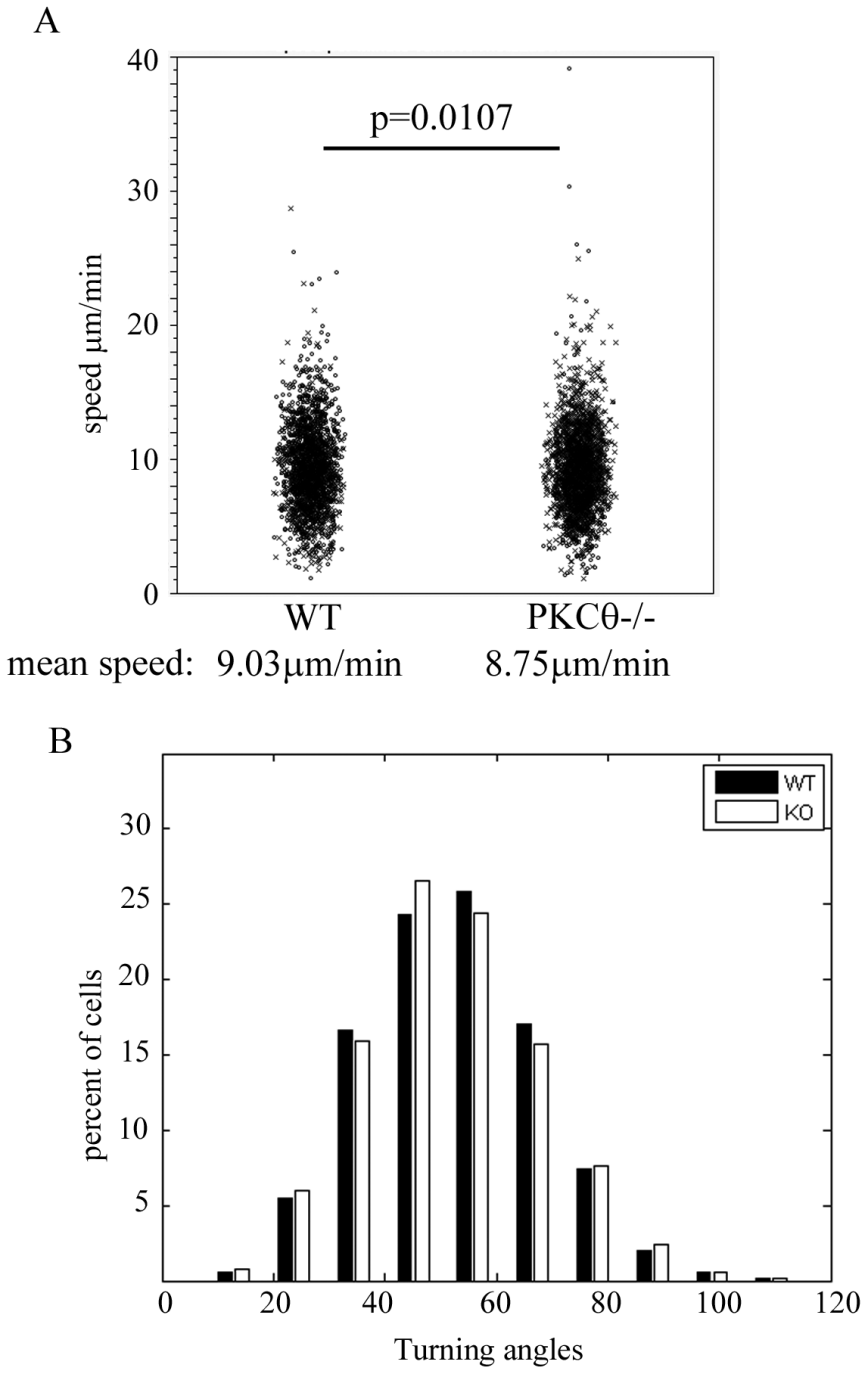
PKCθ plays a role in T cell motility in lymph nodes. WT and PKCθ−/− T cells were isolated and stained with CFSE or CMTMR, injected into recipient mice, and 12–16 hours later, lymph nodes were removed and imaged as described in Materials and Methods using 2-photon microscopy. Cell motility was quantified using Imaris software and (A) speed and (B) turning angles were measured for each cell population. The individual cell mean speed in (A) and mean turning angle (B) are shown. (A) “x” indicates cells dyed with CFSE and “o” shows cells dyed with CMTMR. Statistical significance was calculated using a nested ANOVA analysis (detailed in Materials and Methods).

In addition to migration into lymph nodes, CCL21-CCR7 signaling also regulates T cell migration within lymph nodes [5]. To determine whether PKCθ also has an effect on CCR7 induced intra-lymph node motility, we utilized 2-photon microscopy to visualize T cell migration in intact explanted lymph nodes. We isolated WT and PKCθ−/− T cells, labeling each population with either CFSE or CMTMR, then injected the mixed population into recipient animals. After 12–18 hours, we removed LNs from recipient mice, placed them in a chamber with 95% O_2_/5% CO_2_, then captured images of T cell movement within lymph nodes (Movie S1). We quantified T cell motility and found that PKCθ−/− T cells showed slightly slower speed of movement within lymph nodes compared to WT T cells (Fig. 3A). WT cells moved at a mean speed of 9.03 µm/minute while PKCθ−/− T cells moved at 8.75 µm/min. In addition, we analyzed the turning angles taken by WT and PKCθ−/− T cells but found no significant difference in the mean turning angle (WT T cells: 51.8°; PKCθ−/− T cells 52.4°) or the distribution of the angles taken by WT and PKCθ−/− T cell populations (Fig. 3B).

### PKCθ Regulates Protein Localization to the Uropod and Uropod Length

**Figure 4:**
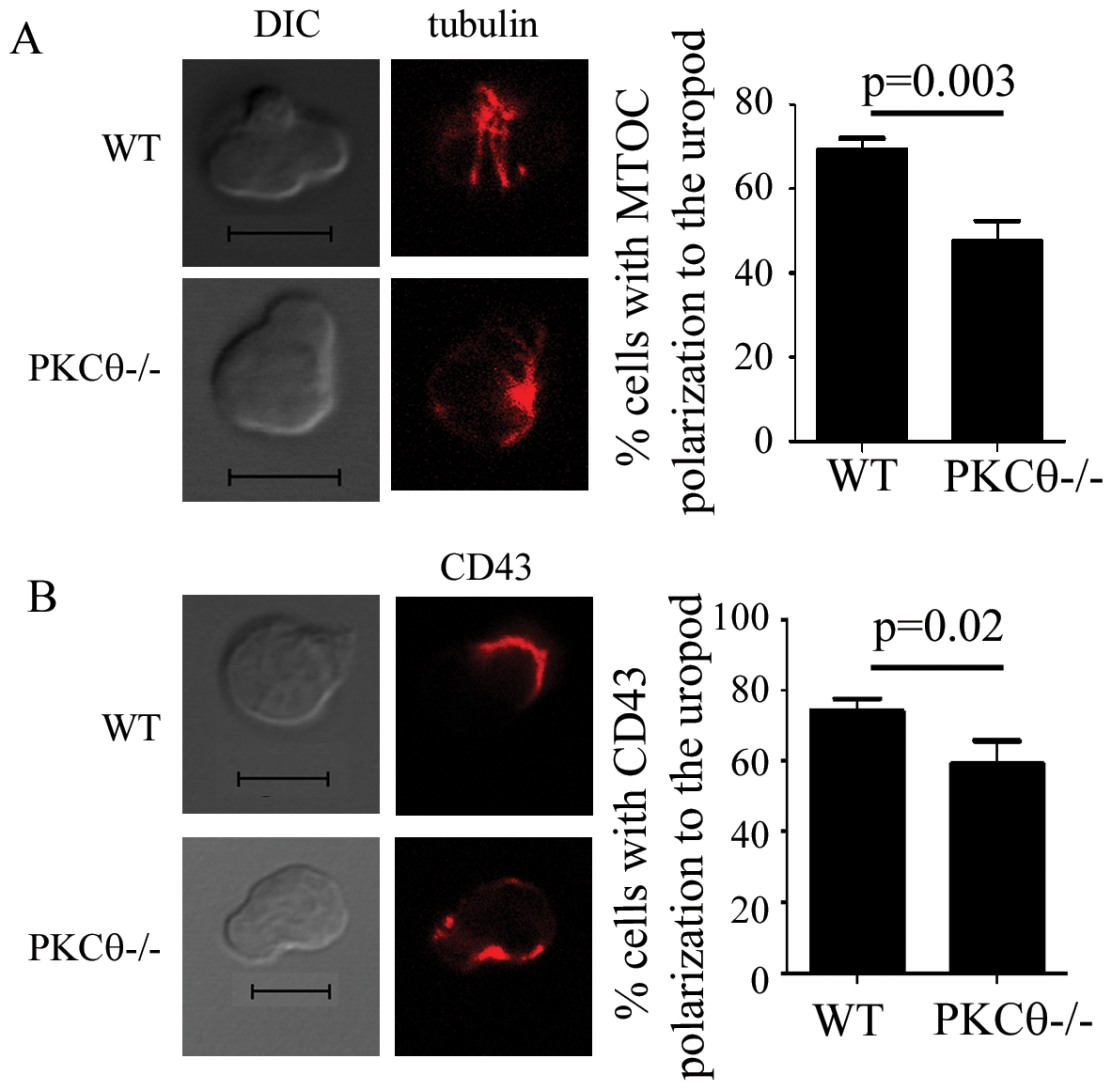
PKCθ regulates MTOC and CD43 localization to uropods. T cells were activated with 300/ml CCL21, fixed, and processed for immunofluorescence. Cells were stained with anti-tubulin (A) in red or anti-CD43 (B). (A) MTOC polarization to the uropod was determined by the presence of the MTOC in the proximal third of the cell including the uropod. For quantitation, at least 50 cells showing T cell uropod morphology were counted for 3 independent experiments, totaling at least 150 cells. Error bars show SEM. Significance was determined using the unpaired student’s t-test.

To understand the mechanism that might underlie the effect of PKCθ on migration to lymph nodes, we determined the effect of PKCθ on uropod formation. Migrating T cell uropods have recently been shown to be crucial for migration into lymph nodes via transendothelial migration, likely through a role in force generation [6], [30]. Uropod formation is regulated in part by specific cytoskeletal protein localization to the uropod which can control both uropod formation and migration [6]. The microtubule organizing center (MTOC), or centrosome, localizes to the T cell uropod to facilitate uropod retraction and T cell motility [27]. Other proteins, including the transmembrane protein CD43, also localize to the uropod [31], [32]. To determine whether PKCθ might affect overall uropod protein localization, we assayed CD43 and MTOC polarization to the uropod in WT and PKCθ−/− T cells activated with CCL21. We found that in the absence of PKCθ, there was a significant decrease in the number of T cells showing MTOC polarization to the uropod (Fig. 4A: WT: 67%; PKCθ−/−: 47.5%) as well as CD43 (Fig. 4B: WT: 73.5%; PKCθ−/−: 55%). These results show that the absence of PKCθ leads to a defect in cytoskeletal organization in the migrating T cell.

**Figure 5:**
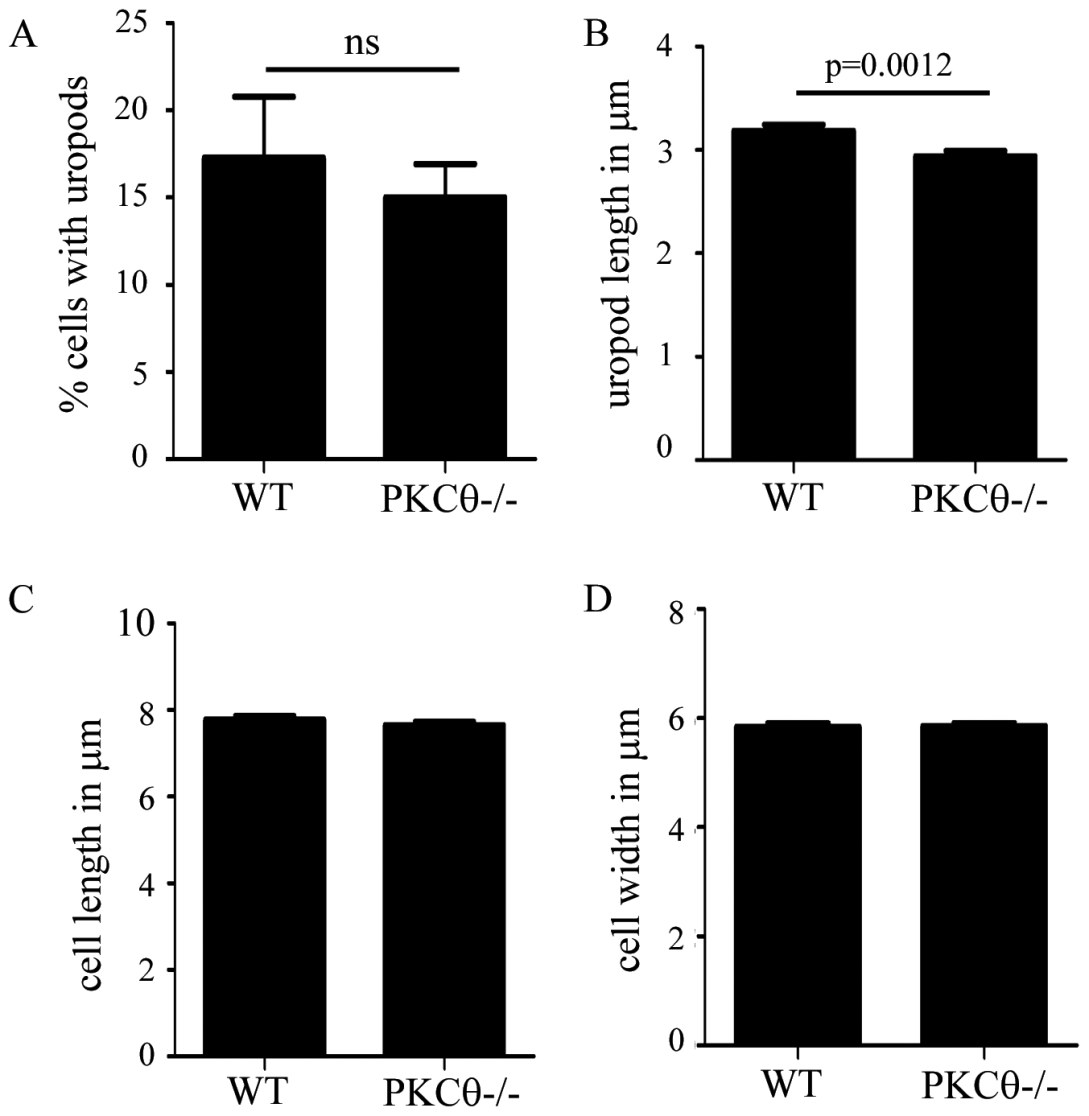
PKCθ regulates uropod length. Isolated T cells were activated with 300/ml CCL21 for 10 minutes and placed onto Poly-L-lysine coated coverslips and fixed. All measurements and quantitation were done using images captured in DIC. (A) Cells were scored as containing a “uropod” if the cell demonstrated the presence of a smaller portion of the cell extending from the main body of the cell. (B) Length of the uropod was determined by measuring the distance from the end of the “uropod” shape to the point where the uropod budded from the main body of the cell using Slidebook. Length (C) and width (D) of the full cell were calculated using Slidebook. The data are from at least 3 independent experiments with at least 50 cells counted from each experiment, totaling at least 150 cells. Significance was determined using the paired student’s t-test.

To determine whether defects in cytoskeletal protein localization resulted in defects in T cell uropod formation, we quantitated uropod formation in PKCθ−/− T cells. We activated purified naïve WT and PKCθ−/− T cells using CCL21, then assessed the migrating T cell for shape, including uropod length. We found no difference between WT and PKCθ−/− T cells in the number of T cells with uropods (Fig. 5A) or total cell length or width (Fig. 5C,D). However, PKCθ−/− T cells did have shorter uropods compared to WT T cells (Fig. 5B, WT 3.2 µm vs. PKCθ−/−2.9 µm). These results suggest that PKCθ can regulate uropod length, possibly through localization of specific cytoskeletal elements including the MTOC.

### PKCθ Affects ERM Protein Localization within Uropods

The Ezrin-radixin-moesin (ERM) family of actin regulatory proteins regulate membrane tension and have been hypothesized to be important in uropod formation [6]. ERM proteins do so by specific localization of proteins to the uropods of migrating T cells [33]. ERM proteins are also responsible for localizing transmembrane proteins including CD43 to this region [8], [10]. PKCθ has been shown to interact with and phosphorylate moesin [34], and as we found a defect in CD43 localization to the uropod, we hypothesized that one potential mechanism by which PKCθ affects T cell uropod formation may be via effects on ERM localization. We asked whether ERM protein localization to the T cell uropod is perturbed in the absence of PKCθ. We purified primary T cells from wild type and PKCθ−/− mice, treated the T cells with CCL21, then fixed and processed the migrating T cells for immunofluorescence. We detected ERM localization in T cell uropods by staining for the ERM family member moesin.

**Figure 6:**
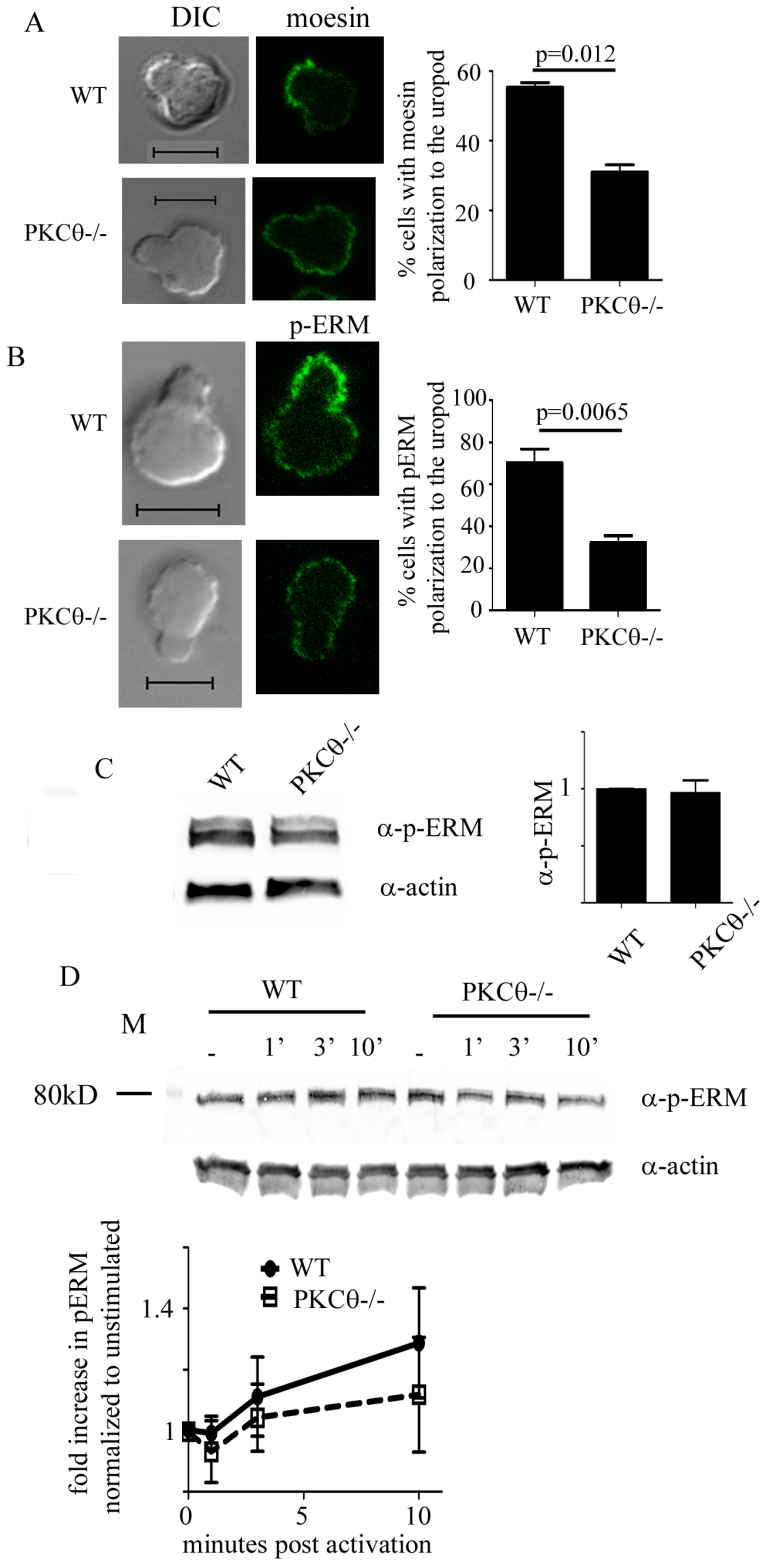
PKCθ is required for ERM phosphorylation and localization to the uropod. T cells were activated with 300/ml CCL21 for 10 minutes, fixed, and processed for immunofluorescence. Cells were stained for moesin (A) or phosphorylated ERM (B). Moesin and p-ERM localization in the uropod was determined measuring intensity in at least 2 spots in and out of the uropod and assessed as “polarized” if the signal in the uropod was at least 50 units or 2-fold higher than that seen outside the uropod. For quantitation, at least 50 cells showing T cell uropod morphology were counted and the localization of MTOC, moesin, or p-ERM assessed in 3 independent experiments, totaling at least 150 cells. Error bars show SEM. Significance was determined using the unpaired student’s t-test. Scale bar indicates 5 µm. (C,D) T cells from C57Bl/6 or C57Bl/6 Ly5.1 (WT) or B6.PKCθ−/− were not activated (C) or activated with 300 ng/ml CCL21 for the indicated time points (D). Cells were lysed and analyzed on SDS–PAGE, transferred onto PVDF membranes and blotted with anti-actin and anti-phospho-ERM antibodies. Signals were quantified using the Licor Odyssey and data shown is representative of at least 3 independent experiments. (C) Fold change in phosphorylated ERM in WT and PKCθ−/− T cells was normalized to the level in the unstimulated WT condition. (D) Fold change in phosphorylated ERM upon CCL21 activation was normalized to the level in unstimulated conditions of either WT or PKCθ−/−.

In agreement with previously published results, we found that a majority of wild type migrating T cells localized moesin to the uropod (Fig. 6A). In contrast, the percentage of T cells showing moesin localization to the uropod was significantly decreased in PKCθ−/− T cells compared to WT T cells (Fig. 6A; WT: 55%; PKCθ−/−: 31%). Because PKCθ has also been shown to phosphorylate moesin, we also determined the phosphorylation state of ERM proteins in the absence of PKCθ. Using an antibody that detects phosphorylation of all members of the ERM family, total level of ERM phosphorylation was not significantly changed in T cells lacking PKCθ in resting T cells (Fig. 6C). Upon treatment of T cells with CCL21, we found that WT cells showed a slight increase in total ERM phosphorylation while PKCθ−/− T cells showed a slightly smaller increase in ERM phosphorylation (Fig. 6D). We also observed cases in which PKCθ−/− T cells showed a slight decrease rather than increase in pERM (gel shown in Fig. 6D). While we saw differences between WT and PKCθ-deficient T cells in phosphorylated ERM levels in response to CCL21, the differences were not statistically significant.

Phospho-ERM also localizes to the uropod, so we assessed phospho-ERM localization in migrating T cells. We found that in WT T cells, p-ERM localized to the uropod in a majority of migrating T cells similar to moesin (WT: 70% Fig. 6B). In contrast, PKCθ−/− T cells were unable to properly localize phosphorylated ERM proteins to the uropod (PKCθ−/−: 32% Fig. 6B). These results show that PKCθ specifically regulates ERM protein localization to the uropod and phosphorylation of ERM proteins at the uropod.

## Discussion

T cell migration is a coordinated process beginning with extracellular chemokine signals leading to integrin activation and T cell motility. In this study, we identify a novel role for PKCθ in regulating T cell motility. Our findings show for the first time that PKCθ can be activated downstream of CCR7 signaling. We find that PKCθ can regulate T cell migration into lymph nodes and motility within lymph nodes. PKCθ affects uropod length and protein localization to the uropod, specifically ERM proteins. As therapeutics targeting PKCθ are being investigated as a treatment for autoimmune diseases, our results shed light on the additional impact that PKCθ may have on T cell migration in addition to the well-documented effects on T cell activation [35].

While many previous studies have implicated the PKC family proteins in the control of cell migration, including T cells, most of these studies used broad non-specific inhibition of PKC family proteins [25], [36], [37]. PKCδ and PKCβ have both been shown to play a role in regulating T cell shape during migration through effects on integrins [26], [29]. Our study provides the first direct evidence for the specific role of PKCθ in regulating T cell motility in response to CCR7 using PKCθ-deficient T cells. We show that PKCθ is activated downstream of CCR7 as demonstrated by increased PKCθ phosphorylation (Fig. 1). While naïve T cells express several chemokine receptors, including CXCR4 and CCR7, CCR7 is uniquely important in driving the homing of cells to lymph nodes, including naïve T cells, dendritic cells, and cancer cells. In T cells and dendritic cells, CCR7 is absolutely required for lymph node trafficking, as defects in either CCR7 or its ligands, CCL21 and CCL19, abolish T cell migration [38], [39]. In addition to our findings, other studies have also shown that PKCθ is also phosphorylated downstream of the chemokine CXCL12, or SDF-1α [40]. Thus, PKCθ activation may be a general feature of chemokine induced motility in T cells.

We find that PKCθ plays a role in regulating in vivo migration of T cells to lymph nodes as well as within lymph nodes. Transendothelial migration requires activation of LFA-1 downstream of CCR7 signaling. In the absence of PKCθ, we saw a defect in homing to lymph nodes (Fig. 2). Our results showing that PKCθ−/− T cells migrate less to LFA-1 suggest that PKCθ acts to regulate entry of T cells into lymph nodes, possibly via LFA-1. In addition, we also find that PKCθ plays a role in intra-lymph node motility (Fig. 3). We saw a small, but statistically significant defect in PKCθ−/− T cell motility within lymph nodes. A previous study had commented that they observed no defect in naïve PKCθ−/− T cell motility in lymph nodes [23], suggesting that the T cell motility defect in PKCθ−/− T cells is not easy to observe in vivo. The magnitude of the defect in PKCθ-deficient T cell migration in vivo reflects the likelihood that multiple PKC proteins including PKCθ, PKCβ,or other PKC family members act in concert to mediate normal T cell migration.

We show for the first time distinct localization of PKCθ and phosphorylated PKCθ within the migrating T cell uropod. Although several PKC family members are expressed in T cells, only PKCθ showed specific localization to the immunological synapse and is the only PKC known to be essential for IL-2 expression [21]. PKCθ function is tightly associated with its localization: PKCθ was the original marker for the immunological synapse (IS) [22]. PKCθ also moves away from the IS between a regulatory T cell and a target cell, suggesting that differential PKCθ localization may be important in controlling effector versus regulatory T cell function [24]. Our results add to the connection between PKCθ localization and function, demonstrating that specific localization of PKCθ within migrating T cells results in effects on T cell uropods and motility.

Our findings point to several potential mechanisms by which PKCθ may regulate T cell motility. We find that PKCθ affects MTOC polarization, which may control T cell retraction, resulting in motility [27]. Defects in MTOC polarization may be responsible for the shortening of uropods in PKCθ−/− T cells (Fig. 4, 5). We also find that PKCθ also regulates ERM and phospho-ERM localization to the uropod (Fig. 6). PKCθ has been previously shown to interact with ERM proteins [41], [42], directly phosphorylating and activating moesin [34]. These studies were done with purified proteins and no direct evidence exists to demonstrate that PKCθ can directly affect ERM phosphorylation in cells. We now show that PKCθ-deficient T cells show a slight decrease in phosphorylated ERM proteins compared to WT cells (Fig. 6). While the difference in in pERM levels between WT and PKCθ−/− T cells is not significant, our results suggest that PKCθ may affect total phosphorylation of ERM proteins as well as ERM localization. Our results differ from published results showing pERM levels decrease upon SDF1α stimulation in human PBCs [43]. Instead of decreasing pERM levels upon SDF1α, we find that WT primary mouse T cells show a slight increase in pERM when stimulated with CCL21. These differences could reflect differences in human and mouse T cells, or signaling downstream of CXCR4 (binding SDF1α) and CCR7 (binding CCL21). Despite these differences, it is clear that chemokine receptor signaling can affect both total pERM levels as well as localization of ERM proteins. Recent evidence shows that ERM proteins directly control integrin function [44] and uropod formation [45] as well as motility within lymph nodes [46]. ERM proteins act on membrane tension in T cells, with constitutively phosphorylated ERM proteins increasing membrane tension [46]. We found that PKCθ−/− T cells showed slightly less ERM phosphorylation, as well as decreased localization of pERM to the uropod, suggesting that PKCθ may affect T cell motility via enhancing ERM phosphorylation and localization to uropods.

We also show PKCθ affects CD43 localization to the migrating T cell uropod (Fig. 4). This is likely to result from defects in ERM protein localization as we have previously shown that ERM proteins are required for CD43 localization [32], [47]. We have also shown that PKCθ can phosphorylate the protein CD43 at a key serine that controls T cell migration, however, we find no defects in CD43 phosphorylation in PKCθ−/− T cells [47], [48] (data not shown).

Several signaling pathways are important for chemokine induced T cell motility, including activation of Rho family GTPases Rac1 and Rap1 via the Rac activator DOCK2 [14], [49], [50]. Another major pathway that regulates signaling from chemokine receptors to cell motility is the PI3K pathway. While the precise contribution of PI3K to neutrophil and T cell migration is still not completely understood [51], [52], [53], it remains to be determined whether PKCθ may intersect with these pathways. In response to TCR signaling, PKCθ has been shown to interact with Akt, the downstream effector of PI3K activation [54]. PKCθ also regulates Rap1 and LFA-1 upon TCR ligation and controls antigen induced migration [23], [55]. Our data demonstrating that T538 and S676 are phosphorylated in response to CCR7 signaling are similar to that seen upon TCR activation [16], [17]. These results suggest that while CCR7 signaling via PKCθ to T cell motility may share some pathways in common with TCR signaling, the effects of PKCθ downstream of CCR7 may be separate from the pathways that have already been identified downstream of TCR signaling.

## Materials and Methods

### Mice

C57BL/6 mice, B6.Ly5.1, and B6.PKCθ-deficient mice were from Jackson Laboratories (Bar Harbor, ME). All mice were bred and/or maintained in a specific pathogen-free condition in barrier facilities (Albuquerque, NM) and conform to the principles outlined by the Animal Welfare Act and the National Institutes of Health guidelines. The protocol was approved by the IACUC at the University of New Mexico (protocol # 10-100487). All efforts were made to minimize suffering.

### Reagents and Antibodies

Antibodies were purchased from the following: α-CD4 was from eBiosciences (San Diego, CA); α-CD62L, and α-CD45.2 were from Biolegend (San Diego, CA); α-actin from Sigma Aldrich (St. Louis, MO); α-tubulin was from Thermo Fisher (Lab Vision, Fremont CA); α-PKCθ, α-phospho-PKCθ S676 was from Santa Cruz Biotechnology (Santa Cruz, CA); α-moesin and α-phospho-PKCθ T538 were from Cell Signaling Technology (Beverly, MA); and α-CD43 antibody S11 was produced in the laboratory of Dr. Anne Sperling at the University of Chicago. CCL21 was from Peprotech (Rocky Hill, NJ), ICAM–Fc from R&D Systems (Minneapolis, MN), CFSE and Calcein-AM were from Invitrogen (Carlsbad, CA), and PKH26 from Sigma Aldrich (St. Louis, MO). For the Li-Cor Odyssey system, α-rabbit 680 conjugates were from Invitrogen, Molecular Probes (Carlsbad, CA) and α-rat 800 conjugates from Rockland Inc. (Gilbertsville, PA). Secondary fluorescently tagged antibodies for immunofluorescence were purchased from Jackson Immunoresearch (West Grove, PA).

### Immunofluorescence Staining and Microscopy

Primary murine T cells were purified by non-adherence to nylon wool, treated and then fixed for 20 min in 3% paraformaldehyde (PFA) in PBS, quenched with 50 mM NH4Cl/PBS, permeabilized for 1 min with 0.3% Triton-X100, and blocked with a PSG solution (PBS, 0.01% saponin, 0.25% aqueous cold fish gelatin, and 0.02% NaN_3_ [all from Sigma, St. Louis, MO]). Fixed cells were incubated with primary antibodies for 1 hour, washed 5 times with PSG, and incubated for 30 minutes with fluorochrome labeled secondary antibodies. Coverslips were washed 5 times with PSG, rinsed with ddH_2_O, and then mounted on slides with Prolong Gold (Invitrogen, Carlsbad, CA). Cells were visualized using a 63×DIC Oil objective on a Zeiss Axioplan 2 MOT upright LSM510 Confocal microscope. Images were obtained using the Zeiss LSM 510 Image Acquisition software and analyzed with the Zeiss LSM Image Browser.

### Two Photon Imaging of Explanted Lymph Nodes

T cells were purified by nylon wool as previously described [32] and purified T cells labeled with either 1 µM CFSE (Invitrogen) or 5 µM CMTMR (Invitrogen). Both WT and PKCθ−/− T cells were labeled with both CFSE and CMTMR to account for dye effects. 5 to 10×10^6^ labeled T cells were injected I.V. into recipient mice and inguinal lymph nodes were removed 15–18 hours later and imaged using two photon-imaging.

Imaging experiments were performed using a workstation with a Bio-Rad Radiance 2000 scanner mounted on an Olympus upright microscope with a chamber at 37°C. Explanted lymph nodes were placed on a glass cover-slip in the chamber. The sample is perfused with a 37°C solution of DMEM (phenol red free, Gibco) bubbled with 95% O_2_ and 5% CO_2_. T cell behavior within a lymph node was monitored in the T cell area at a minimum of 70 µm below the surface of the node. For 4D analysis of T cell motility, multiple stacks in the z axis (z step = 3 µm) were acquired every 15–20sec (depending on the number of z stacks acquired) for 15–40 min, with an overall field thickness of 40–60 µm. Cell motility was analyzed with Imaris software (version 6; Bitplane). Tracks that lasted fewer than 3 time steps (duration filter in Imaris) were not taken into account in the analysis. Length filter (threshold of 17 µm = 3 times the diameter of the cell) Displacement^2^ filter (threshold of 300 µm^2^ = 17 µmX17 µm) were also used to discard tracks of non-motile cells. Videos were made by projecting the 4D information along the z axis in a single plane.

### Immunoblotting

Nylon non-adherent lymph node T cells were treated with either 50 ng/ml PMA and 500 ng/ml ionomycin or 300 ng/ml CCL21for the indicated times, and lysed in lysis buffer (0.5% TX-100, 150 mM NaCl, 50 mM Tris pH 7.6, 5 mM EDTA, 1 mM NaF, 1 mM Na_3_VO_4_, and protease inhibitors: 10 µg/ml aprotinin, 1 mM Pefabloc (from Roche Applied Sciences, Mannheim, Germany), and 10 µg/ml leupeptin), before being analyzed by SDS–PAGE. All western blotting signal was detected with the Odyssey system (Li-Cor Biosciences, Lincoln, NE). Quantitation was done by drawing a region of interest around the protein of interest, and all levels of proteins of interest were normalized to the level of actin within the same sample through detection of both proteins simultaneously. Statistical significance in changes in phosphorylated PKCθ and ERM proteins was determined by comparing the levels of phosphorylation at each time point with the baseline phosphorylation using the paired student’s t-test.

### In Vivo Migration

Competitive migration assays were performed as described [47]. Nylon wool non-adherent primary murine T cells were labeled with either 0.5 µM CFSE or 0.5 µM PKH-26. Differentially dyed populations were mixed in equal numbers, and 5–10×10^6^ cells injected into recipient B6.Ly5.1 mice. Ly5.2 (CD45.2) and CD4+ populations were gated and % CFSE and PKH26 populations determined and compared. Analyses were performed using Flowjo (Treestar Inc. Ashland OR).

### Transwell Migration Assay

1×10^5^ T cells were labeled with 5 µM CFSE or 0.25 µM CFSE, mixed, and added in a 1∶1 ratio to the top of a Costar (Corning Acton, MA) 3.0 µm Transwell permeable support apparatus. For CCL21 conditions, 300 ng/ml CCL21 was added to the bottom of the transwell apparatus. For ICAM conditions, transwell apparatus was coated with 6 µg/ml ICAM overnight, washed in PBS, then blocked with 2.5% BSA for 2 hours, washed, and cells added to the top. At the end of the incubation period, transwell apparatuses and unmigrated cells were discarded, and migrated cells analyzed and normalized to the input population using the LSRII (BD Biosciences, San Jose, CA).

### Statistical Analysis

All statistics except for data captured via 2-photon microscopy were done using an unpaired or paired Student two-tailed *t* test as indicated in figure legends. Error bars represent SEM. For intra lymph node cell motility data, mean speed of individual cells and turning angles taken at each time step were quantified. For statistical analysis, we used nested ANOVA and found that there was no statistically significant effect of the field of cells, the lymph node, the mouse, or the date of experiment performed. We also used nested ANOVA to statistically control the effect of the dye on each cell population. We used the ANOVA test to determine statistical difference in mean speed and turning angle in Figure 3 when controlling for the effect of the dye on WT and PKCθ−/− T cell populations.

## Supporting Information

Movie S1 **In vivo motility of WT and PKCθ−/− T cells in intact lymph nodes.** WT and PKCθ−/− T cells were purified and stained with CFSE (WT) and CMTMR (PKCθ−/−), injected into recipient mice, and 12–18 hours later, inguinal lymph nodes from recipient animals were removed and imaged using 2-photon microscopy as described in the materials and methods.
(AVI)
